# Efficacy of moxibustion in diabetes peripheral neuropathy

**DOI:** 10.1097/MD.0000000000028173

**Published:** 2021-12-10

**Authors:** Jing Sheng Tay, Yun Jin Kim

**Affiliations:** School of Traditional Chinese Medicine, Xiamen University Malaysia, Jalan Sunsuria, Bandar Sunsura, Sepang, Selangor, Malaysia.

**Keywords:** diabetes peripheral neuropathy, moxibustion, randomized controlled trial

## Abstract

**Background:**

Diabetic peripheral neuropathy (DPN) is one of the most common complications of diabetes mellitus. The main clinical manifestations of DPN include pain, numbness, paraesthesia, and weakness of the lower limbs which often leads to diabetic foot ulceration, eventually resulting in amputation. Based on Traditional Chinese Medicine theory, moxibustion has a great effect on treating and preventing DPN. However, randomized clinical trials done to evaluate the efficacy of this treatment are still lacking. Hence, this study is carried out to evaluate the effectiveness and safety of moxibustion therapy on diabetic peripheral neuropathy.

**Methods:**

This study will be a pilot, interventional, randomized, 2-armed, parallel, singled-masked, controlled trial. A total of 40 diabetes mellitus patients with peripheral neuropathy will be recruited and assigned randomly into 2 groups (moxibustion group and waiting group) at a 1:1 ratio. This trial consists of an 8-week intervention period and a 4-week follow-up period. During the intervention period, the moxibustion group will take 3 moxibustion sessions per week, whereas no intervention will be done on the waiting group to act as the control group. The outcome will be assessed by an outcome assessor who is unaware of the group assignment. The primary outcome will be pain assessment measured with algometry, Leeds Assessment of Neuropathic Symptoms and Signs pain scale, visual analogue scale, and neuropathy pain scale. The secondary outcome will be an evaluation of functional performance capacity with 6 minutes walking test, evaluation of the Foot and Ankle Ability Measure, and serum HbA1c and albumin levels.

**Discussion:**

We hope that this trial will provide valuable insights on the efficacy of moxibustion in the management of diabetic peripheral neuropathy.

**Trial registration:**

ClinicalTrials.gov Registry No.: NCT04894461 (URL: https://clinicaltrials.gov/ct2/show/NCT04894461?term=NCT04894461&draw=2&rank=1) Registered on May 20, 2021.

## 1 Introduction

Diabetic peripheral neuropathy (DPN) is one of the most common complication of diabetes mellitus patients, with prevalence of 50.7% in Malaysia.^[1]^ It is a crucial factor that can lead to the development of diabetic foot ulceration and is one of the major reasons of nontraumatic lower extremity amputations in many of the high-income nations.^[2]^ The main clinical manifestations of DPN includes pain, numbness, paresthesia, and weakness of the lower limbs, and patients can easily injure their foot unaware, which leads to ulceration, eventually resulting in amputation.^[3]^ Considering how serious the complications can get, DPN should be diagnosed as soon as possible and treat accordingly. However, the efficacy of the treatment is not promising.^[4]^

Many physicians have initiated study to explore how Traditional Chinese Medicine (TCM) can help in DPN therapy. Based on TCM theory, moxibustion can regulate qi and blood, warm meridian, and improve blood circulation, which may help treat and prevent DPN.^[4]^ TCM external treatment are commonly utilized in clinical practice. For instance, acupuncture has been proven to be therapeutically beneficial and is frequently used to treat DPN.^[5,6]^ Moxibustion, on the other hand, was showed in some studies to be able to raise serum superoxide dismutase level,^[7]^ lower free-radical production, protect nerve tissues from damage due to free-radical build-up, and relieve neuro-inflammation, potentially through inhibition of NF-κB and activation of Nrf2.^[8]^ Liu et al^[9]^ found out that moxibustion with methylcobalamin is more effective than using methylcobalamin only, also Li^[10]^ showed that treatment using methylcobalamin and alpha lipoic acid can be more effective when combined with moxibustion therapy. Lan et al^[3]^ study showed that moxibustion can help reduce the Toronto clinical neuropathy score and simple pain scale score (VAS) of DPN patients. Nevertheless, randomize clinical trial done to evaluate the efficacy of moxibustion treatment is still lacking, especially when compared to other treatment like acupuncture therapy.^[11]^

The primary aim of this study is to evaluate the effectiveness and safety of moxibustion therapy on diabetes peripheral neuropathy.

## 2 Methods

### 2.1 Objective

The main objective of this pilot, interventional, randomized, 2-armed, parallel, singled-masked, controlled trail study is to evaluate the clinical efficacy and safety of moxibustion single treatment in DPN patients.

### 2.2 Study design and setting

**Figure 1:**
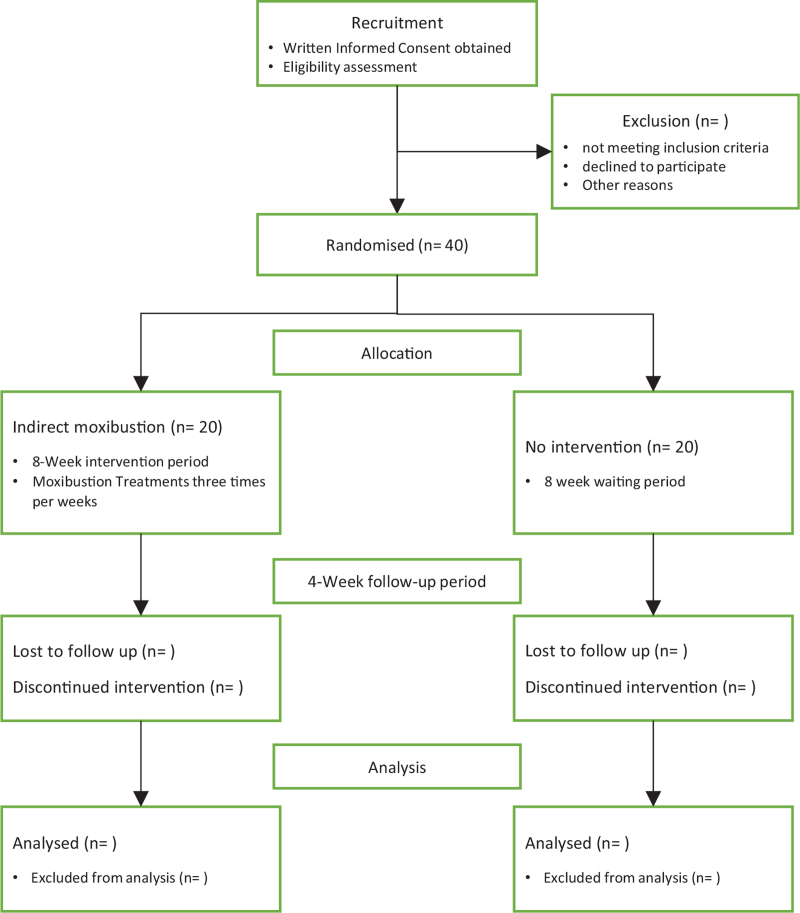
Flowchart of the clinical trial.

This study is designed as a pilot, interventional, randomized, 2-armed, parallel, singled-masked, controlled trial. This study will be conducted at TCM Skill Training Centre, Xiamen University Malaysia from November 2021 to December 2023. A total of 40 diabetes mellitus patients with peripheral neuropathy will be recruited to participate in this trial and will be assigned randomly into 2 groups (moxibustion group and waiting group) at 1:1 ratio. The outcome assessor in this study will be unaware of the group assignments. This trial consists of 8-week intervention period and 4-week follow-up period. The study design is illustrated in a flow chart in Figure 1. Present clinical trial study has not completed participant recruitment at the time of submission.

### 2.3 Ethics approval and consent to participation

The protocol was approved by the institutional review board of School of Traditional Chinese Medicine, Xiamen University Malaysia, where the protocol study will take place [Approved ID: TCM/2021/IRB/02]. This clinical trial was registered with the ClinicalTrials.gov Registry No.: NCT04894461 (https://clinicaltrials.gov/ct2/show/NCT04894461?term=NCT04894461&draw=2&rank=1) on May 19, 2021. The clinical trial will be monitored by independent data and safety control. All participants will be providing written informed consent before recruitment.

### 2.4 Participants

#### 2.4.1 Recruitment

Recruitment will be started on November 1, 2021 (the anticipated date of first enrolment will be November 1, 2021, and the recruitment period may be extended depending on registration during the COVID-19 pandemic status in the Malaysia). A consent document that contains information about the background, purpose, moxibustion intervention, clinical outcome and benefits, and adverse effects will be given to the all participants or their legal surrogates. The investigating clinical researchers will explain the consent details before enrolling the clinical trials and notify all subjects about the option to withdraw without any penalty during the clinical trials.

#### 2.4.2 Inclusion criteria

Participants who meet the following criteria will be considered eligible to participate in the clinical study. The inclusion criteria are:

1. Age between 18 and 75 years.
2. Diagnosed with diabetes mellitus, according to World Health Organization’ criteria.
3. Proven to have peripheral neuropathy.
4. Documented consent to participate in this study and acknowledgement that all relevant information is received.
5. Willing and able to meet the planned visit and meet the planned schedule, including participation in the experimental investigations.

#### 2.4.3 Exclusion criteria

Participants who meet ≥1 of the following criteria will not be considered eligible to participate in the clinical study. The exclusion criteria are:

1. Having medical or surgical diseases unsuitable to participate in this study, as assessed by health professionals.
2. Pregnancy or breastfeeding (for female participants).
3. Having other competitive conditions that can cause peripheral neuropathy.
4. Participants involved in the planning or execution of this study.
5. Having severe cognitive impairment and difficulty to understand the clinical trial protocol.
6. Unable to receive moxibustion treatment due to any reasons.

### 2.5 Discontinuation criteria

Participants are encouraged to complete all study evaluations, they may withdraw from the clinical study at any time and for any reason. Every effort will be made to determine why any participants withdraw from the study prematurely. This information should be recorded. If a participant prematurely, all data normally collected at discharge should be collected at the time of premature discontinuation or the scheduled discharge. Participants may be terminated before completing the clinical study for any of the following discontinuation criteria are:

1. Participants have serious adverse reactions during the clinical trials and it is not suitable to continue participating in this study.
2. Participants have serious clinical complications or deteriorating medical conditions during the clinical trials.
3. Participants ask to terminate the trial halfway.
4. Participants show poor compliance and cannot comply with the clinical trial protocol.

### 2.6 Recruitment and randomization

Who signed the inform consents participants will be subjected to the measurement of vital signs (eg, blood pressure, pulse rate, and body temperature), demographic information survey, medical history (eg, past, present, and family), and physical examination. Eligible participants who meet the inclusion and exclusion criteria will receive a schedule of clinical trials.

The randomization at a 1:1 ratio into the 2 study arms will be performed via a computer-generated randomization list (prepared by SAS Visual Data science Decisioning, SAS Institute Inc., Cary, NC), which will be kept at the TCM Skill Training Center, Xiamen University Malaysia. Randomization will be stratified by the TCM Skill Training Centre. This computer-generated randomization list show only one result at a time and is unavailable to the enrolling study clinical researcher.

### 2.7 Study procedure

At visit 1, the study clinical researcher will check the participants’ vital signs, medical history, physical examination, algometry pain assessment, the Leeds assessment of neuropathic symptoms and signs (LANSS) pain scale, VAS, the Neuropathy Pain Scale (NPS), functional performance capacity with 6 minutes walking test, the foot and ankle ability measure (FAAM), and laboratory tests (includes, serum HbA1c, and serum albumin levels). The intervention group will received moxibustion management for 30 minutes. In the meantime, the clinical researcher will check the participants’ adverse effects.

From visit 2 to 24 (8 weeks), the intervention group will be treated the same manner as visit 1, and clinical researcher will be check the participants’ algometry pain assessment, the LANSS pain scale, VAS, the NPS, functional performance capacity with 6 minutes walking test, the FAAM, and laboratory tests (includes, serum HbA1c, and serum albumin levels) on visit 12 and visit 24.

At visit 25 (follow-up), the clinical researcher will check the participants’ adverse effects and measured the same manner as visit 12 and visit 24.

If any additional visits may be performed at any time by the clinical researcher's judgment, or following the participant's request. In the event of an unexpected visit, the following tests and assessment will be performed: vital signs, checking adverse events, and laboratory tests. The detailed study period is shown in Table 1.

**Table 1:**
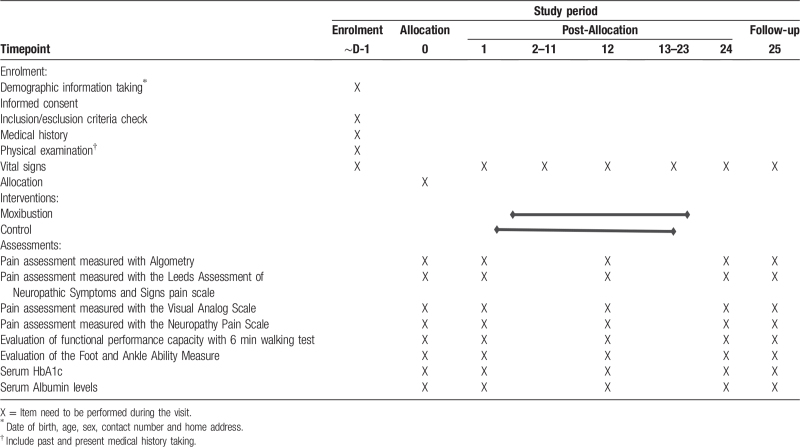
The study period of clinical trials.

### 2.8 Intervention

#### 2.8.1 Intervention group

After randomization, patients of intervention group will receive moxibustion treatment provided by registered physician at TCM Skill Training Centre, Xiamen University Malaysia. The clinical researchers will perform indirect moxibustion on Hegu (LI4), Fenglong (ST40), Quchi (LI11), Sanyinjiao (SP6), Zusanli (ST36), and Taichong (LR3). The treatment session will take 30 minutes per session, and will be done for 3 times per week, lasting for 8 weeks. All moxibustion management was performed by an experienced TCM practitioners in the School of Traditional Chinese Medicine, Xiamen University Malaysia, and also registered practitioners in the Division of Traditional & Complementary Medicine, Ministry of Health Malaysia.

#### 2.8.2 Control group

No intervention will be conducted on the control group.

### 2.9 Outcomes

#### 2.9.1 Primary outcome measure

Pain assessment measured with Algometry: It is used to determine sensitivity to pain produced by pressure.

Pain assessment measured with the LANSS: The LANSS Pain Scale is based on analysis of sensory description and bedside examination of sensory dysfunction, and provides immediate information in clinical settings.^[12]^ The possible scores range from 0 to 24, with a score of ≥12 considered to be suggestive of neuropathic pain.

Pain assessment measured with the VAS: A VAS is a measurement instrument that tries to measure a characteristic or attitude that is believed to range across a continuum of values and cannot easily be directly measured. It is often used in epidemiologic and clinical research to measure the intensity or frequency of various symptoms.

Pain assessment measured with the NPS: Scores are based on patient responses to questions about pain intensity. 0 indicates no pain; 10 indicates the most pain imaginable. The NPS is only for use in patients who have already been diagnosed with neuropathic pain.^[13]^

#### 2.9.2 Secondary outcome measure

Evaluation of functional performance capacity with 6 minutes walking test: It is a sub-maximal exercise test used to assess aerobic capacity and endurance. The distance covered over a time of 6 minutes is used as the outcome by which to compare changes in performance capacity. It is plays a key role in measure functional exercise capacity, assessing prognosis and evaluating response to treatment.^[14]^

Evaluation of the FAAM: It is a self-report outcome instrument developed to assess physical function for individuals with foot and ankle related impairments. It is a 29-item questionnaire divided into two subscales: the FAAM, 21-item Activities of Daily Living Subscale and the FAAM, 8-item Sports Subscale. Each item is scored on a 5-point Likert scale (4 to 0) from “no difficulty at all” to “unable to do.” Item score totals which range 0 to 84 for the Activities of Daily Living subscale and 0 to 32 for the Sports subscale were transformed to percentage scores. A higher score represents a higher level of function for each subscale.^[15,16]^

Serum HbA1c: It provides accurate long-term index of average blood glucose level.

Serum albumin levels: It measures the amount of the protein albumin in human blood.

### 2.10 Safety evaluation

Study clinical researchers will ask the participants at each visit to identify if they have experienced any adverse effects (AEs). Participants will also be asked to report AEs voluntarily. Any AEs cases will be documented with details on occurrence, duration, severity, and how it is resolved. The case will also be categorized as treatment-related or not treatment-related. Common treatment-related AEs include allergies, burns, infection, coughing, nausea, vomiting, and so on.

### 2.11 Data collection and management

Data will be extracted from the online case reports form and questionnaires. All data will be recorded in the online case reports form stored in the TCM Skill Training Center, Xiamen University Malaysia password-protected computer. At least 2 data administrators will log in the information independently and proofread it.

## 3 Data analysis

SPSS will be used in statistical analysis. Continuous variable will be shown as mean ± SD, while categorical variables will be shown as percentages and numbers. The significance will be calculated using Student *t* test for matching data or Wilcoxon-test depending on whether normality can be assumed or not. *P* values <.05 will be considered as significantly different.

## 4 Discussion

DPN is the most prevalent and problematic complication of diabetes mellitus, with the highest rate of morbidity and mortality, as well as a significant financial burden on diabetes treatment.^[17]^ Controlling hyperglycemia and maintaining a steady blood glucose level is currently the main important method for preventing and treating DPN. Other treatment options for DPN, including those used in clinical trials, have demonstrated poor success, particularly for nonpainful symptoms.^[18]^

The sensory nerve conduction velocityof a patient can be reduced by DPN, which can affect sensation and movement. Through acupoint therapy, sensory nerve conduction velocity can be increased and DPN symptoms can be relieved.^[19]^ Electroacupuncture and acupoint injections with mecobalamin at Shenshu (BL23) and Zusanli (ST36), for example, have been shown to enhance the expression of nerve growth factor and its receptor TrkA.^[20]^ Similarly, catgut implantation in Zusanli (ST36), Pishu (BL20), and Shenshu (BL23) together with taking TCM prescription “Tangtong Drink,” increases nerve growth factor levels while reducing serum transforming growth factor-1 levels.^[21]^ They both help in nerve regeneration and the healing of nerve damage. In addition, acupuncture or acupoint injection can raise superoxide dismutase expression^[22]^ and content of nitric oxide in the serum,^[23]^ which both have beneficial effects on neuropathy patients. However, high level of high-sensitivity C-reactive protein (hs-CRP), malondialdehyde, and homocysteine is associated with causing or aggravating neuropathy.^[24]^ Researchers found that salvia injections at Zusanli (ST36) combined with lipoid acid intravenous injection inhibit the expression of hs-CRP and lowers malondialdehyde levels.^[22]^ Another study showed low-frequency electroacupuncture at Zusanli (ST36), Zhongwan (CV12), and Mingmen (DU4) combined with mecobalamin injection can lower hs-CRP and homocysteine levels,^[24]^ thus beneficial in treating DPN patients.

Traditional Chinese medicine believes that DPN belongs to the category of “*bi* syndrome,” which is a blockage of meridians due to pathogenic factors including wind, cold, phlegm, and dampness, resulting malnutrition of meridian and blood stasis.^[25]^ This can be treated via acupunctures, moxibustion, or other techniques that can dredge meridians. There are several researchers who have studied the effectiveness of acupuncture in treating DPN, but researches in the effectiveness of moxibustion is lesser.^[11]^ Both acupunctures and moxibustion can influence the flow of qi in the meridian via stimulation of acupoints, but moxibustion technique can be less invasive and safer, which can be important in treating DPN patients as they usually have impaired wound healing. Patient may also be able to perform moxibustion themselves at home, which may be a benefit as it brings convenience to the patient.

## 5 Conclusion

In this study, it is impossible to blind the physician performing the moxibustion, and thus we tried to minimize potential bias by blinding the outcome assessor. High-quality data will be collected from this randomized, 2-arm parallel, controlled trial to determine whether moxibustion is effective for DPN. We hope that this trial will provide valuable insights on the efficacy of moxibustion on the management of DPN (Supplementary Content).

## Author contributions

JST: analyzed and interpreted the patient data, and was a major contributor in writing the manuscript. YJK: designed the study plan, methods, materials, and was a major contributor in writing the manuscript. All authors read and approved the final manuscripts.

**Conceptualization:** Jing Sheng Tay, Yun Jin Kim.

**Data curation:** Jing Sheng Tay, Yun Jin Kim.

**Formal analysis:** Jing Sheng Tay.

**Funding acquisition:** Yun Jin Kim.

**Investigation:** Jing Sheng Tay, Yun Jin Kim.

**Methodology:** Jing Sheng Tay, Yun Jin Kim.

**Project administration:** Jing Sheng Tay, Yun Jin Kim.

**Supervision:** Yun Jin Kim.

**Validation:** Jing Sheng Tay, Yun Jin Kim.

**Writing – original draft:** Jing Sheng Tay, Yun Jin Kim.

**Writing – review & editing:** Jing Sheng Tay, Yun Jin Kim.

## Supplementary Material

Supplemental Digital Content
