# Supplementary material for: Efficacy of moxibustion in diabetes peripheral neuropathy
Source: Medicine (Baltimore). 2021 Dec 10;100(49):e28173. doi: 10.1097/MD.0000000000028173 (PMC8663870; doi:10.1097/MD.0000000000028173)
Supplement: Supplemental Digital Content [file medi-100-e28173-s001.doc]

**School of Traditional Chinese Medicine**

**Xiamen University Malaysia**

**Efficacy of Moxibustion in Diabetes Peripheral Neuropathy: Clinical Trials**

**(XMUMRF-C6/ITCM/0004)**

**INFORMED CONSENT STATEMENT**

You are invited to participate in a research study.

**Background and Introduction**

Diabetic peripheral neuropathy (DPN) is one of the most common complication of diabetes mellitus patients, with prevalence of 50.7% in Malaysia. It is a crucial factor that can lead to the development of diabetic foot ulceration and is one of the major reasons of non-traumatic lower extremity amputations in many of the high-income nations. The main clinical manifestations of DPN includes pain, numbness, paraesthesia, and weakness of the lower limbs, and patients can easily injure their foot unaware, which leads to ulceration, eventually resulting in amputation. Considering how serious the complications can get, DPN should be diagnosed as soon as possible and treat accordingly. However, the efficacy of the treatment is not promising.

**Study objective**

The main objective of this study is to evaluate the clinical efficacy and safety of Moxibustion single treatment in DPN patients.

**Clinical Trials Title**

Efficacy of Moxibustion in Diabetes Peripheral Neuropathy: Clinical Trials.

**Study Plan**

This study is designed as a pilot, interventional, randomised, 2-armed, parallel, singled-masked, controlled trial. This study will be conducted at TCM Skill Training Centre, Xiamen University Malaysia from November 2021 to December 2023. A total of 40 diabetes mellitus patients with peripheral neuropathy will be recruited to participate in this trial and will be assigned randomly into 2 groups (moxibustion group and waiting group) at 1:1 ratio. The outcome assessor in this study will be unaware of the group assignments. This trial consists of 8-week intervention period and 4-week follow-up period.

At visit 1, the study clinical researcher will check the participants’ vital signs, medical history, physical examination, algometry pain assessment, the Leeds assessment of neuropathic symptoms and signs (LANSS) pain scale, visual analogue scale (VAS), the neuropathy pain scale (NPS), functional performance capacity with 6 minutes walking test, the foot and ankle ability measure (FAAM), and laboratory tests (includes, serum HbA1c, and serum albumin levels). The intervention group will received moxibustion management for 30 minutes. In the meantime, the clinical researcher will check the participants’ adverse effects.

From visit 2 to 24 (8 weeks), the intervention group will be treated the same manner as visit 1, and clinical researcher will be check the participants’ algometry pain assessment, the Leeds assessment of neuropathic symptoms and signs (LANSS) pain scale, visual analog scale (VAS), the neuropathy pain scale (NPS), functional performance capacity with 6 minutes walking test, the foot and ankle ability measure (FAAM), and laboratory tests (includes, serum HbA1c, and serum albumin levels) on visit 12 and visit 24.

At visit 25 (follow-up), the clinical researcher will check the participants’ adverse effects and measured the same manner as visit 12 and visit 24.

If any additional visits may be performed at any time by the clinical researcher’s judgment, or following the participant’s request. In the event of an unexpected visit, the following tests and assessment will be performed: vital signs, checking adverse events, and laboratory tests.

**Risks and Emergency Medical Treatment**

Study clinical researchers will ask the participants at each visit to identify if they have experienced any adverse effects (AEs). Participants will also be asked to report AEs voluntarily. Any AEs cases will be documented with details on occurrence, duration, severity, and how it is resolved. The case will also be categorized as treatment-related or not treatment-related. Common treatment-related AEs include allergies, burns, infection, coughing, nausea, vomiting, and so on. The clinical researcher notify participants that you could make decision whether you continue to participate in the clinical trials. Further medical consultation or referral to Klinik Salak Tinggi will be attange, if necessary.

**Benefits**

Research leads to many advances in diagnosis and treatment of disorders. Taking part in this clinical trials may not only benefit to you individually, but also helps to find a treatment for DPN. However, if positive results could be obtained from this trials, your participation is no doubt beneficial to you and other patients in the future. Free charges for acupuncture treatment will be provided to you.

**Responsibilities**

If you agree to participate in this clinical trials, you need to provide your personal information, including name, age, address, telephone number, email address and medical history. You will be instructed to eat the same number of meals regularly..

**Confidentiality**

All information only for research used and no personal information would be released.

**Contact**

If you have questions at any time about the study or the procedures, you may contact our management office.

Principle investigator: Dr. Yun Jin Kim

Office Telephone number: +603-87055098

If you feel you have not been treated according to the descriptions in this form, or your right as a participant in research have been violated during the course of this project, you may contact the Committee on the Use of School of Traditional Chinese Medicine, Xiamen University Malaysia,

**Participation**

Your participant in this clinical trials is voluntary; you may decline to participate without penalty. If you decide to participate, you may withdraw from the study at any time without penalty and without loss of benefits to which you are otherwise entitled. If you withdraw from the study before data collection is completed your data will be returned to you or destroyed.

**Consent**

I have read and understand the above information. I have received a copy of this form. I agree to participate in this study.

Participant Signature______________________________Date _______________________

Principle Investigator's Signature_____________________Date _______________________

*** This form can be accepted by digital signature during COVID-19 pandemic periods in Malaysia.*
